# Heartburn or angina? Differentiating gastrointestinal disease in primary care patients presenting with chest pain: a cross sectional diagnostic study

**DOI:** 10.1186/1755-7682-2-40

**Published:** 2009-12-12

**Authors:** Stefan Bösner, Jörg Haasenritter, Annette Becker, Maren A Hani, Heidi Keller, Andreas C Sönnichsen, Konstantinos Karatolios, Juergen R Schaefer, Erika Baum, Norbert Donner-Banzhoff

**Affiliations:** 1 Department of General Practice/Family Medicine, University of Marburg, 35032 Marburg, Germany; 2 Department of Family Medicine, Paracelsus University, 5020 Salzburg, Austria; 3 Department of Cardiology, University of Marburg, 35032 Marburg, Germany

## Abstract

**Background:**

Gastrointestinal (GI) disease is one of the leading aetiologies of chest pain in a primary care setting. The aims of the study are to describe clinical characteristics of GI disease causing chest pain and to provide criteria for clinical diagnosis.

**Methods:**

We included 1212 consecutive patients with chest pain aged 35 years and older attending 74 general practitioners (GPs). GPs recorded symptoms and findings of each patient and provided follow up information. An independent interdisciplinary reference panel reviewed clinical data of each patient and decided about the aetiology of chest pain. Multivariable regression analysis was performed to identify clinical predictors that help to rule in or out the diagnosis of GI disease and Gastroesophageal Reflux Disease (GERD).

**Results:**

GI disease was diagnosed in 5.8% and GERD in 3.5% of all patients. Most patients localised the pain retrosternal (71.8% for GI disease and 83.3% for GERD). Pain worse with food intake and retrosternal pain radiation were associated positively with both GI disease and GERD; retrosternal pain localisation, vomiting, burning pain, epigastric pain and an average pain episode < 1 hour were associated positively only with GI disease. Negative associations were found for localized muscle tension (GI disease and GERD) and pain getting worse on exercise, breathing, movement and pain location on left side (only GI disease).

**Conclusions:**

This study broadens the knowledge about the diagnostic accuracy of selected signs and symptoms for GI disease and GERD and provides criteria for primary care practitioners in rational diagnosis.

## Background

Chest pain is a common complaint in primary care. 20 to 40% of the population will visit their GP for chest pain during their lifetime [1]. While incidence varies according to setting, country and inclusion criteria [2-4], there is a similar range of different diseases that can cause chest pain in a primary care setting[5,6]. Gastrointestinal (GI) aetiologies have been quoted with frequencies of 8%-17% and constitute an important aetiology for chest pain encountered by the General Practitioner (GP) [3-7].

GPs face the challenge to diagnose serious cardiac disease reliably but also to identify other causes including appropriate testing and treatment.

There is still a lack of data on the presentation of GI disease in chest pain patients derived from larger samples in a primary care setting. Our prospective study aims to describe the distinctive clinical characteristics of GI disease and Gastroesophageal Reflux Disease (GERD) and to give support for rational diagnosis in a primary care setting.

## Methods

We conducted a cross-sectional diagnostic study with a delayed-type reference standard in a primary care setting [8]. The final diagnosis was established by an expert panel after 6 months of follow up. The aim of the initial study was to investigate the diagnostic accuracy of signs and symptoms for chest pain patients with Coronary Heart Disease (CHD). In this article we report results of a secondary analysis in regards to the accuracy of clinical criteria for GI disease and GERD.

### Participating GPs and patients

We approached 209 GPs in the State of Hesse of whom 35.4% agreed to participate in the study. Only GPs being prepared to undergo random recruitment quality controls could take part. Participating practices had to recruit consecutively every attending patient who had chest pain either as presenting complaint or on questioning. The recruitment period lasted 12 weeks for each practice. For logistical reasons recruitment was staggered in four waves between October 2005 and July 2006.

Every patient above 35 years with pain localized in the area between clavicles and lower costal margins and anterior to the posterior axillary lines was to be included. Doctors were also asked to recruit at home visits and emergency calls. Patients were eligible irrespective of the acute or chronic nature of their complaint, or of previously known conditions including Coronary Heart Disease (CHD) or related risk factors. However, patients whose chest pain had subsided for more than one month, whose current episode of chest pain had been already investigated resulting in a definite diagnosis and/or who came for follow-up investigations for chest pain (e.g. an orthopaedic doctor had already established a diagnosis and the GPs continues the treatment) were excluded. In emergency situations without sufficient time for patient information and written consent, relevant clinical items were documented on a case report form (CRF) kept by the GPs. Later, e.g. after discharge from hospital, the patient was asked to participate in the study. Only if he gave informed consent the CRF was handed over to study personnel. Like the whole study protocol, this procedure was approved by the Ethics Committee of the Faculty of Medicine, University of Marburg. The study complies with the declaration of Helsinki.

### Data collection

GPs took a standardized history and performed a physical examination according to a CRF that was piloted and modified accordingly. We chose determinants based on results of qualitative interviews conducted with GPs on how they diagnose chest pain. The CRF contained items about patient (e.g. patients known to the GP, patient different as usual) and pain characteristics (time, duration, location and radiation), accompanying symptoms (e.g. dyspnoea, cough, vomiting), risk factors for CHD, results of a basic physical examination and the GP's presumed diagnosis. GPs also recorded their preliminary diagnoses, investigations and management related to the patients' chest pains. Patients were contacted by phone six weeks and six months after the index consultation. Study assistants blinded to clinical data that already had been recorded asked about the course of their chest pain, treatments including hospitalisations and drugs. Discharge letters from specialists and hospitals were requested from GPs.

### Precautions against bias

Participating practices were recruited from a network of research practices associated with our department. To GPs we emphasized the importance of recruiting every patient with chest pain, irrespective of the presumed likelihood of CHD. Practices were visited at four week intervals to check CRFs, recruitment logs and compliance with study procedures. Random quality controls were performed by searching routine documentation of participating practices to identify cases of chest pain not included in the study.

### Reference standard

A reference panel consisting of one cardiologist, one GP and one research staff of the department of Family Medicine reviewed baseline and follow up data of each patient. As far as possible the definition and diagnostic recommendations of the guideline "Reflux" of the German society for digestive and metabolic diseases were taken into account. However, investigations as suggested by the guideline were often not performed by GPs. Therefore, data available to the reference panel were often limited. Apparently the diagnosis of GERD was made on the basis of the history alone. Our 'delayed-type reference standard' may provide some reassurance against alternative causes, e.g. peptic ulcer or carcinoma, of participating patients' complaints. The GP's initial diagnosis contributed to the decision made by the panel.

### Statistical analysis

Our main analysis for distinctive clinical characteristics associated with GI disease and GERD is based on the sample of all patients with chest pain where diagnostic classification was possible. For univariate analyses we calculated separately for GI disease and GERD diagnostic odds ratios (OR) for all items covered by the CRF. To arrive at a small subset of criteria for clinical recommendation we selected those index test items that had a p-value < 0.05 and ORs indicating at least moderate diagnostic accuracy, i.e. OR+/- >2 or <0.5 (univariate analysis). Those were included as predictive variables in multivariate logistic regression analysis. The outcome variables were GI disease and GERD. Variable selection was conducted using the backward stepwise procedure (p < 0.05). Odds ratio and 95%-confidence intervals were calculated. All analyses were performed with SPSS software version 14.0.

## Results

### GP and general patient characteristics

**Figure 1:**
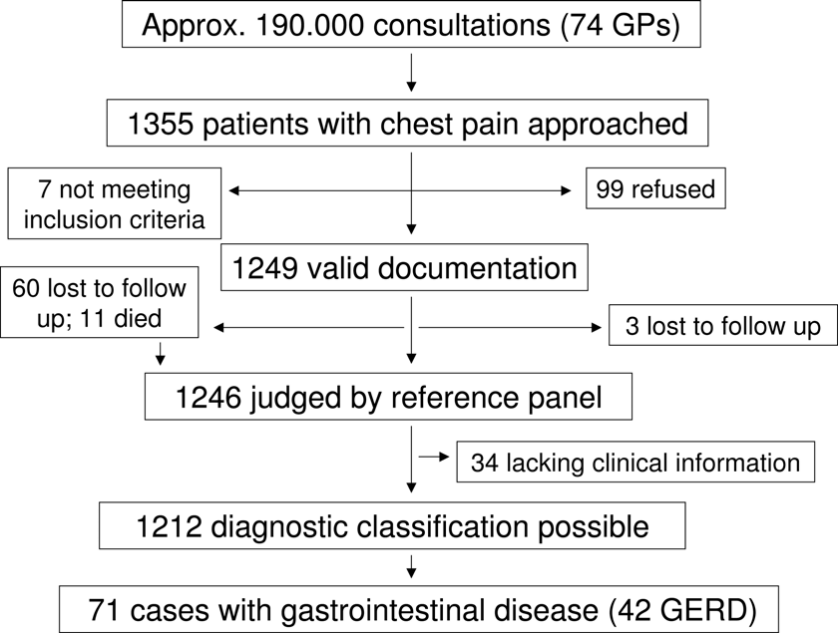
**Patient recruitment**.

The majority of participating GPs were male (67%), two thirds of practices were located in urban areas (63.5%). Mean age of GPs was 49 years. According to our estimate these 74 GPs encountered around 190.000 patients during the study period and approached 1355 patients with chest pain. 7 patients did not meet the inclusion criteria after review of patient records by the study investigators and were consequently excluded; 99 refused to participate in the study. 60 cases were lost to follow up and 11 died but provided enough information to be judged by the reference committee; 3 cases were early drop outs and were therefore not included. For 34 cases follow-up information was lacking, incomplete or ambiguous so that no final diagnosis could be made. We thus analysed 1212 patients for the aetiology of their chest pain; of these 71 were diagnosed as having GI disease including 42 patients with GERD (figure 1).

The prevalence of chest pain during the study period was 0.7% (CI: 0.66-0.73) of all patient contacts. Major diagnostic groups were: GI disease 5.8% (95% CI: 4.5% - 7.1%), Chest Wall Syndrome 46.6% (95% CI: 43.8% - 49.4%), Ischemic Heart Disease (stable) 11.1% (95% CI: 9.4% - 12.8%), psychogenic disorders 9.5% (95% CI: 7.9% - 11.3%) and upper respiratory infections 8.1% (95% CI: 6.6% - 9.8%).

Gastroesophageal reflux disease (GERD) alone contributed with 3.5% (95% CI: 2.6% - 4.7%) (n = 42), benign stomach problems with 2.1% (95% CI: 1.5% - 3.1%) (n = 26) and diseases of the biliary tract with 0.3% (CI: 0.1% - 0.7%) (n = 3) to the aetiology of chest pain.

Compared with CHD patients, patients with GI disease and GERD tend to have a lower mean age and a higher proportion of females. While around half of the patients with GI disease had chest pain at the time of consultation, this was only the case with one third of CHD and GERD patients. In comparison to the other 2 groups, patients with GERD were presenting in a lower proportion with acute, i.e. duration <48 hours, chest pain (table 1).

**Table 1:** Basic characteristics of CHD patients, patients presenting with GI disease including GERD and with GERD

Basic characteristics	Patients with CHD (n = 180)	Patients with GI disease (n = 71)	Patients with GERD (n = 42)
Mean age (range)	69 years (35 - 91 years)	58 years (35 - 86 years)	58 years (35 -86 years)

Sex - female patients - n (%)	88 (48.9)	47 (66.2)	25 (59.5)

Chest pain at the time of consultation - n (%)	56 (31.1)*	33 (47.1)*	15 (35.7)*

Known by the GP - n (%)	167 (92.8)	65 (91.5)	39 (92.9)

Chest pain as reason for consultation - n (%)	149 (82.8)	64 (90.1)	37 (88.1)

Acute chest pain (< 48 hours) - n (%)	51 (28.3)*	19 (27.9)*	7 (16.7)*

* Slightly changing denominator because of missing data

### Clinical characteristics

26.8% of patients with GI disease and 19.0% with GERD complained of vomiting. In 21.1% (GI disease) and 21.4% (GERD) pain was worse with food intake. 49.3% of patients with GI disease and 51.2% with GERD thought that their chest pain would be of cardiac origin.

For GI disease pain was localised retrosternal in 71.8% (60.5%-80.9%), epigastric in 29.6% (20.2%-41.0%), left in 32.45 (22.7%-43.9%) and right in 23.9% (15.55-35.0%). For GERD retrosternal location was 83.3% (69.4%-91.7%) and epigastric location was 19.2% (10.0%-33.3%).

**Figure 2:**
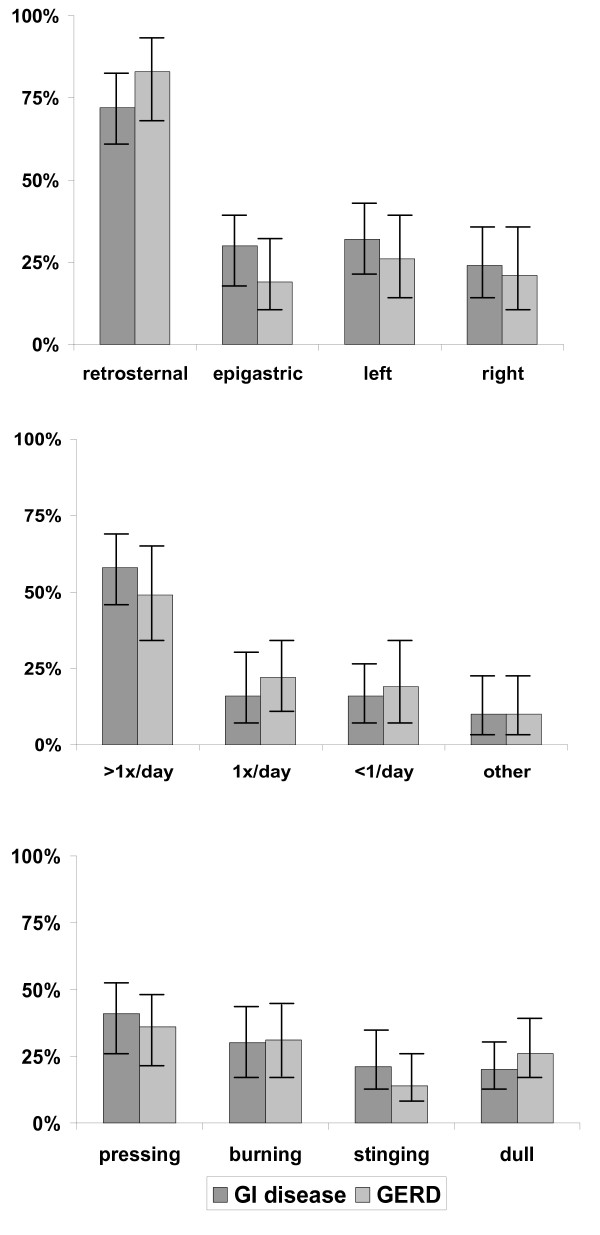
**Pain localisation, frequency and character of patients presenting with GI disease (n = 71) and with GERD (n = 42)**.

The majority of patients reported a pain frequency of more than one episode per day (58.2% (46.3%-69.3%) for GI disease, 48.8% (34.3%-63.5%) for GERD). Pain character was perceived by most patients as either pressing (40.8% (30.2%-52.5%) for GI disease, 35.7% (23.0%-50.8%) for GERD) or burning (29.6% (20.2%-41.0%) for GI disease, 30.9% (19.1%-46.0%) for GERD). Pain characteristics are listed in figure 2.

### Follow up of patients with GI disease and GERD

71.8% of patients with GI disease and 71.4% of patients with GERD still complained of chest pain when contacted after 6 weeks by telephone. After a follow up period of 6 months these numbers were reduced to 47.9% and 52.4% respectively. 74.6% of patients with GI disease and 85.7% of patients with GERD continued to consult their GP during the follow up period related to the initial problem of chest pain.

### Univariate and multivariate analysis

15 items (listed in the footnote of table 2) fulfilled our univariate selection criteria for GI disease and GERD and were selected for multivariable analysis the results of which are reported in table 2.

**Table 2:** Clinical characteristics associated with GI disease (including GERD) and with GERD (multivariable model, n = 1212)

Index test	Unadjusted OR (95%-CI)	adjusted OR (95%-CI)	p value
**Association with GI disease**			

Burning pain	3.30 (1.92-5.67)	3.16 (1.53-6.49)	<0.01

Vomiting,	5.59 (3.14-9.97)	8.01 (3.80-16.85)	<0.01

Pain worse with food intake	18.83 (8.86-40.02)	31.27 (8.82-110.82)	<0.01

Retrosternal pain localisation	1.95 (1.15-3.32)	2.37 (1.10-5.10)	0.03

Epigastric pain localisation	2.48 (1.45-4.25)	3.48 (1.63-7.46)	<0.01

Retrosternal pain radiation	6.68 (3.51-12.72)	5.02 (1.90-13.25)	<0.01

Average pain episode < 1 hour	2.34 (1.36-4.02)	3.30 (1.57-6.91)	<0.01

Pain worse with movement,	0.22 (0.10-0.52)	0.36 (0.14-0.94)	0.04

Pain depending on exercise	0.21 (0.08-0.57)	0.15 (0.04-0.58)	<0.01

Localised muscle tension	0.45 (0.26-0.80)	0.44 (0.22-0.90)	0.03

Pain worse with breathing	0.21 (0.07-0.57)	0.33 (0.11-0.99)	0.05

Pain on the left side	0.25 (0.15-0.42)	0.42 (0.22-0.81)	0.01

**Association with GERD**			

Pain worse with food intake	14.23 (6.09-33.27)	16.49 (4.39-61.99)	<0.01

Retrosternal pain localisation	3.83 (1.69-8.71)	3.18 (0.98-10.32)	0.05

Retrosternal pain radiation	8.29 (3.93-17.49)	6.32 (2.21-18.06)	<0.01

Pain on the left side	0.19 (0.10-0.39)	0.40 (0.14-1.11)	0.08

Localised muscle tension	0.25 (0.10-0.61)	0.35 (0.13-0.94)	0.04

For GI disease the following variables were selected for multivariable analysis (according to performance markers in univariate analysis: p < 0.05; OR >2 or <0.5): burning pain, vomiting, pain worse with ingestion, retrosternal pain localisation, epigastric pain localisation, pain on the left side, retrosternal pain radiation, average pain episode < 1 hour, average pain episode < 1 minute, pain worse with movement, pain depending on exercise, stinging pain, localized muscle tension, patient dyspnoeic, pain worse with breathing.

For GERD the following variables were selected for multivariable analysis (according to performance markers in univariate analysis: p < 0.05; OR >2 or <0.5): burning pain, vomiting, pain worse with ingestion, retrosternal pain localisation, pain on the left side, retrosternal pain radiation, continuous pain, average pain episode < 1 hour, pain worse with movement, pain depending on exercise, stinging pain, localized muscle tension, pain worse with breathing, pain at the time of consultation, pain reproduced by palpation.

Pain worse with food intake and retrosternal pain radiation were associated positively with both GI disease and GERD. Retrosternal pain localisation, vomiting, burning pain, epigastric pain and an average pain episode < 1 hour were associated positively with GI disease. Negative associations were found for localized muscle tension (GI disease and GERD) and pain depending on exercise, pain worse with breathing or movement, pain on the left side (GI disease).

In table 3 we present for clinical use diagnostic items that were significant in the multivariable analysis with corresponding likelihood ratios (LR). These can help to rule in or to rule out GI disease and GERD [9].

**Table 3:** Clinical recommendation

**Useful for including disease**	**LR +**	**Useful for excluding disease**	**LR +**
			
**GI disease**			
Pain worse with food intake	21.00	Pain depending on exercise	0.27
Vomiting	4.50	Pain worse with breathing	0.27
Retrosternal pain	5.25	Pain worse with movement	0.29
**GERD**			
Pain worse with food intake	10.5	Localized muscle tension	0.38
Retrosternal pain radiation	6.39		

## Discussion

Among patients presenting with chest pain in general practice, GI disease is with 5.8% (including 3.5% GERD) the fifth common aetiology. Pain worse with food intake and retrosternal pain radiation were associated positively with both GI disease and GERD; retrosternal pain localisation, vomiting, burning pain, epigastric pain and an average pain episode < 1 hour were associated positively only with GI disease.

There are strengths and limitations to this study. To our knowledge this is the largest study investigating the epidemiology and aetiology of chest pain in primary care. Patients were consecutively recruited in a large number of urban and rural practices. The participating GPs' demographic characteristics are similar to the population of GPs in the State of Hesse (data available upon request). Study procedures, including random quality controls, reduced the possibility of selection bias.

We did not interfere with the work-up provided by participating GPs. As a result of this, only limited data of GI investigations were available to the reference panel. Given the difficulty in organising research in general practice settings, we consider our design as appropriate. Due to the broad range of diseases that can cause chest pain we could not standardise case definitions for all single diseases. Case definition of GI and GERD is based on clinical findings and the GP's presumed diagnosis and was not systematically investigated by a gastroscopy. Therefore misclassifications cannot be excluded. However, existing guidelines were used as much as possible and planning a delayed diagnosis improved the accuracy of the reported diagnosis.

As clinical findings and the GP's presumed diagnosis (data from the original questionnaire) were also used by the panel for decision making there is a certain degree of incorporation bias.

Other studies conducted in a primary care setting quote higher frequencies of GI aetiologies (8%-17%) for chest pain [3-6]. One possible explanation is different inclusion/exclusion criteria. A Swedish study group excluded all patients with known CHD; a higher relative aetiological contribution of other diseases might have resulted from this exclusion [3]. Work up bias and different reference standards might be an additional explanation as studies differed in the length of the follow up period and the way a final diagnosis was decided on [5,7].

GPs think in the diagnostic work up process of patients rather in pragmatic than in distinct aetiological disease categories. We therefore analysed our data for two reference parameters, GI disease (including GERD, benign stomach disease, and diseases of the biliary tract) and GERD alone. Predominant symptoms for GERD are heartburn, a burning pain situated in the retrosternal area, and regurgitation of gastric acid [10-13]. In our study one third of GERD patients complained of burning pain and 72% localised the pain in the retrosternal region.

There are only few studies in regards to the diagnostic utility of reflux disease symptoms [14]. Two studies conducted in a different setting (gastroenterological clinic) examined in a consecutive and prospective study design diagnostic characteristics of selected symptoms for GERD in patients with non-cardiac chest pain [15,16]. Burning pain showed a sensitivity of 57% and positive LR of 2.7[16] which is in line with our findings for GI disease (positive LR of 2.7). However, in our sample burning pain did not discriminate significantly for GERD alone. Davies et al. examined patients with acute chest pain in an emergency setting for oesophageal aetiologies. They found positive LRs of 10.1 for 'pain triggered by ingestion', 3.7 for heartburn, 2.0 for vomiting and 1.2 for retrosternal pain localisation [17]. We also found all above mentioned symptoms being of significant diagnostic accuracy for GI disease and partly for GERD.

When encountering patients with chest pain, the GP faces the problem that single symptoms might not yield enough diagnostic information to base a sound decision on. The clinician therefore has to combine different clinical symptoms and signs [18]. Whereas in our study single likelihood ratios for each sign and symptom were at best moderate, they contribute to rule in or out GI disease or GERD when applied in succession [9]. A positive proton pump inhibitor (PPI) test (at least 50% clinical improvement after prescription of a high dosed PPI for 2-4 weeks) can be an additional aid for the diagnosis of GERD. However, two metaanalyses showed only moderate sensitivity (80%) and specificity (74%) for patients with non-cardiac chest pain[19,20] and these results have to be interpreted with caution when considering to apply them for a primary care population.

## Conclusions

In conclusion, GI disease including GERD is the fifth most frequent cause of chest pain in primary care. This study provides data on the diagnostic accuracy of selected signs and symptoms which will help primary care practitioners to narrow the likelihood of GI disease.

## Competing interests

JRS acts as scientific advisor for MSD and ESSEX. All other authors do not declare any competing interests.

## Authors' contributions

NDB formulated the research question, designed the study and supervised its conduct together with ACS. NDB, EB, JH, AB, ACS, MAH, HK, JRS, KK and SB were involved in acquisition, analysis and interpretation of data (MAH, HK in data acquisition, NDB, JRS, ACS, KK, SB in interpretation of data of the reference panel, NDB, EB, AB, JH, SB in data analysis). SB drafted the article. All authors read and approved the final manuscript.
